# Polar vasculosis is associated with better kidney outcome in type 2 diabetes with biopsy‐proven diabetic kidney disease: A multicenter cohort study

**DOI:** 10.1111/jdi.14059

**Published:** 2023-07-22

**Authors:** Miho Shimizu, Kengo Furuichi, Tadashi Toyama, Masayuki Yamanouchi, Junichi Hoshino, Shinji Kitajima, Akinori Hara, Yasunori Iwata, Norihiko Sakai, Yukio Yuzawa, Hiroshi Kitamura, Hiroshi Sato, Yugo Shibagaki, Yoshiki Suzuki, Noriko Uesugi, Yoshihiko Ueda, Kentaro Kohagura, Kenichi Samejima, Kazuhiko Tsuruya, Shinichi Nishi, Tomoya Nishino, Hirofumi Makino, Seiichi Matsuo, Yoshifumi Ubara, Hitoshi Yokoyama, Takashi Wada, Research Group of Diabetic Nephropathy, the Ministry of Health, Labour and Welfare, and the Japan Agency for Medical Research and Development

**Affiliations:** ^1^ Department of Nephrology and Laboratory Medicine, Graduate School of Medical Sciences Kanazawa University Kanazawa Japan; ^2^ Department of Nephrology Kanazawa Medical University Uchinada Japan; ^3^ Nephrology Center Toranomon Hospital Tokyo Japan; ^4^ Department of Nephrology Tokyo Women's Medical University Tokyo Japan; ^5^ Department of Hygiene and Public Health, Graduate School of Medical Sciences Kanazawa University Kanazawa Japan; ^6^ Fujita Health University Toyoake Japan; ^7^ Department of Pathology National Hospital Organization Chiba‐Higashi National Hospital Chiba Japan; ^8^ JR Sendai Hospital Sendai Japan; ^9^ Division of Nephrology, Department of Internal Medicine St Marianna University School of Medicine Kawasaki Japan; ^10^ Division of Clinical Nephrology and Rheumatology Niigata University Medical and Dental Hospital Niigata Japan; ^11^ Department of Pathology Fukuoka University Fukuoka Japan; ^12^ Department of Pathology Dokkyo Medical University Saitama Medical Center Koshigaya Japan; ^13^ Dialysis Unit University of the Ryukyus Hospital Nakagami‐gun Japan; ^14^ Department of Nephrology Nara Medical University Kashihara Japan; ^15^ Division of Nephrology and Kidney Center Kobe University Graduate School of Medicine School of Medicine Kobe Japan; ^16^ Department of Nephrology Nagasaki University Hospital Nagasaki Japan; ^17^ Okayama University Okayama Japan; ^18^ Nagoya University Nagoya Japan

**Keywords:** Diabetic kidney disease, Kidney outcome, Polar vasculosis

## Abstract

**Aims/Introduction:**

This multicenter cohort study retrospectively assessed the association between polar vasculosis and the progression of diabetic kidney disease (DKD) in type 2 diabetes.

**Materials and Methods:**

We enrolled 811 patients with type 2 diabetes, biopsy‐proven DKD, and proteinuria (≥0.15 g/g creatinine [g/day]). The association between polar vasculosis and other kidney lesions was explored. The outcome was DKD progression defined as a composite of renal replacement therapy initiation or 50% decline in estimated glomerular filtration rate (eGFR) from baseline.

**Results:**

Of the 811 cases, 677 (83.5%) had polar vasculosis. In multivariate logistic regression analysis, subendothelial widening of the glomerular basement membrane, glomerulomegaly, glomerular class in the Renal Pathology Society classification ≥IIb, vascular lesions, age, eGFR, and hemoglobin A1c were positively associated with polar vasculosis, whereas interstitial fibrosis and tubular atrophy (IFTA) was negatively associated with polar vasculosis. During a median follow‐up of 5.2 years, progression of DKD occurred in 322 of 677 (7.4 events/100 person‐years) and 79 of 134 (11.4 events/100 person‐years) cases with and without polar vasculosis, respectively. Kaplan–Meier analysis showed that polar vasculosis was associated with lower cumulative incidences of DKD progression. Multivariate Cox regression analyses showed that polar vasculosis was associated with a lower risk of DKD progression, regardless of eGFR or proteinuria subgroups. These associations between polar vasculosis and better kidney outcome were unchanged considering all‐cause mortality before DKD progression as a competing event.

**Conclusions:**

This study showed that polar vasculosis of DKD was associated with less advanced IFTA and a better kidney outcome in type 2 diabetes with proteinuria.

## Graphical abstract

This multicenter cohort study retrospectively assessed 811 proteinuric patients with type 2 diabetes and biopsy‐proven diabetic kidney disease (DKD) in Japan to investigate the association between polar vasculosis and DKD progression. This study showed that polar vasculosis of DKD was associated with less advanced interstitial fibrosis and tubular atrophy and a better kidney outcome in type 2 diabetes patients with proteinuria.

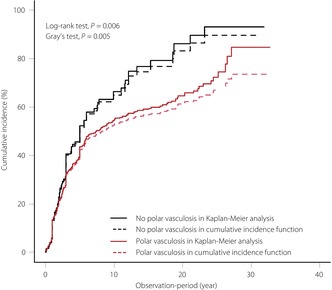

## INTRODUCTION

The clinical course and outcomes in diabetic kidney disease (DKD) are heterogeneous and various phenotypes of DKD exist, but DKD is the leading cause of end‐stage kidney disease globally, including Japan^1^. Clinical trials have demonstrated that medications, such as the use of renin–angiotensin–aldosterone system (RAAS) inhibitors and sodium–glucose cotransporter (SGLT) 2 inhibitors, exert renoprotective effects^2^. However, kidney lesions associated with a favorable kidney outcome have not been reported.

Abnormal angiogenesis is involved in the pathogenesis of DKD, and the development of extra vessels, termed polar vasculosis (perihilar neovascularization), is noted as an early kidney lesion in DKD^3^, ^4^, ^5^, ^6^, ^7^, ^8^, ^9^, ^10^, ^11^, ^12^. Although the exact structure of polar vasculosis is currently unknown, a study using reconstruction of three‐dimensional images suggests that these vessels are angiogenesis connecting afferent arterioles, anastomosed glomerular capillaries, efferent arterioles, and peritubular capillaries^4^. These extra vessels have features such as a thinned basement membrane and enlarged endothelial cells, often with hyalinosis, and are also observed in the glomerular tuft, Bowman's capsule, and the peritubular areas^3^, ^5^.

Although the function of polar vasculosis is also unknown, it has been speculated that the formation of polar vasculosis may decrease the intraglomerular pressure^7^, ^10^. We reported previously on the clinical and pathological features that affect the speed of declining kidney function in patients with biopsy‐proven diabetic nephropathy^12^. In that report, we demonstrated that polar vasculosis is associated with a preserved eGFR for 3 years after kidney biopsy^12^. However, studies investigating the association between polar vasculosis and long‐term kidney outcome in DKD are scarce. We therefore investigated the associations of polar vasculosis with other kidney lesions, clinical characteristics, and long‐term kidney outcome in patients with type 2 diabetes, biopsy‐proven DKD, and proteinuria.

## METHODS

### Study design and participants

This is a retrospective study of patients who underwent clinical kidney biopsy after obtaining informed consent and had a pathological diagnosis of DKD between June 1981 and January 2020 at the following 14 hospitals in Japan: Kanazawa University Hospital (Ishikawa, Japan), Kanazawa Medical University Hospital (Ishikawa, Japan), Toranomon Hospital (Tokyo, Japan), Fujita Health University Hospital (Aichi, Japan), National Hospital Organization Chiba‐East National Hospital (Chiba, Japan), Tohoku University Hospital (Miyagi, Japan), St Marianna University School of Medicine Hospital (Kanagawa, Japan), Niigata University Hospital (Niigata, Japan), University of Tsukuba Hospital (Ibaraki, Japan), Nara Medical University Hospital (Nara, Japan), Kobe University Hospital (Hyogo, Japan), Nagasaki University Hospital (Nagasaki, Japan), Okayama University Hospital (Okayama, Japan), and Nagoya University Hospital (Aichi, Japan). Kidney biopsy was performed on patients with type 2 diabetes in whom it was necessary to confirm clinically precise pathological findings. The exclusion criterion was patients with other kidney diseases, except for nephrosclerosis coexisting with DKD. Among 1,050 patients with type 2 diabetes and biopsy‐proven DKD, 93, 30, 30, and 86 patients were excluded for lack of data on urinary protein excretion, lack of data on the presence or absence of polar vasculosis, lack of data on kidney outcome, and baseline proteinuria <0.15 g/g creatinine [g/day], respectively. Thus, 811 patients were included in the final analysis. This study was approved by the Kanazawa University Ethics Committee [No. 2016‐460 (1204)] and all participating institutions.

### Clinical examinations

The following parameters at the time of kidney biopsy (baseline) were collected by manual chart review: age, sex, serum creatinine, eGFR, urine protein‐to‐creatinine ratio, 24 h urinary protein excretion, hemoglobin A1c, systolic and diastolic blood pressure, total cholesterol, use of antihyperglycemic medications, use of RAAS inhibitors, use of calcium channel blockers, and use of lipid‐lowering agents. eGFR was calculated based on the equation established by the Japanese Society of Nephrology^13^. According to the classification of chronic kidney disease (CKD), baseline eGFR was classified as G1–G2 (≥60 mL/min/1.73 m^2^) and G3a–G5 (<60 mL/min/1.73 m^2^) for categorical analyses^14^. According to the revised CKD classification by the Japanese Society of Nephrology, baseline urinary protein excretion was categorized as 0.15–0.49 g/g creatinine (g/day) and ≥0.5 g/g creatinine (g/day)^14^, ^15^. Levels of hemoglobin A1c were presented according to the standards of National Glycohemoglobin Standardization Program and International Federation of Clinical Chemistry. Hypertension was defined as systolic blood pressure ≥140 mmHg and/or diastolic blood pressure ≥90 mmHg, irrespective of antihypertensive medication use.

### Pathological examinations

Biopsy samples were stained using periodic acid–Schiff, periodic acid methenamine–silver, hematoxylin–eosin, and Mallory–Azan (or Masson's trichrome), and subsequently examined under a light microscope. The definition and scoring of all pathological lesions were agreed upon after reviewing previous pathological studies on diabetic nephropathy and after more than 2 years of meetings among the authors. Furthermore, we published the “Manual for pathological diagnosis of diabetic nephropathy and hypertensive nephrosclerosis”, focusing on the characteristic lesions of diabetic nephropathy/DKD and the common lesions of diabetic nephropathy/DKD and hypertensive nephrosclerosis in 2014^1^, ^16^. In the process of developing our manual before the study^1^, ^16^, we examined the same biopsy specimens and discussed them among ourselves in order to reduce inter‐observer variability and to improve consistency in the scoring. Based on previous studies that used this manual, the scoring system in this study was as follows: nine glomerular lesions (polar vasculosis [yes/no]; diffuse mesangial expansion [grade 0–3]; nodular sclerosis [yes/no]; subendothelial widening of glomerular basement membrane [GBM; grade 0–3]; exudative lesion [yes/no]; mesangiolysis [yes/no]; ratio of global glomerulosclerosis to the total number of glomeruli; ratio of segmental glomerulosclerosis to the total number of glomeruli; and glomerulomegaly [yes/no]), two tubulointerstitial lesions (interstitial fibrosis and tubular atrophy (IFTA) [grade 0–3] and interstitial inflammation [grade 0–3]), and two vascular lesions (arteriolar hyalinosis [grade 0–3] and arteriosclerosis [grade 0–2])^1^, ^12^, ^16^, ^17^, ^18^. We also classified glomerular lesions based on the pathological classification of diabetic nephropathy, proposed by the Renal Pathology Society (RPS)^19^. Nephrologists and/or pathologists examined the specimens at each participating institution.

### Outcome

The outcome was the progression of DKD, defined as a composite of renal replacement therapy initiation or 50% decline in eGFR from baseline. Data were obtained from the medical records of all participating institutions. Cases that did not achieve these outcomes or were lost to follow‐up were censored at the last follow‐up visit.

### Statistical analysis

Data are expressed as median and interquartile ranges for continuous variables and percentages for categorical variables, which were compared using the Mann–Whitney *U* test and Fisher's exact test, respectively. To determine the factors associated with the presence of polar vasculosis, univariate and multivariate logistic regression analyses were performed including 12 pathological variables in the Japanese classification^1^, ^16^ (diffuse mesangial expansion, nodular sclerosis, subendothelial widening of GBM, exudative lesion, mesangiolysis, global glomerulosclerosis, segmental glomerulosclerosis, glomerulomegaly, IFTA, interstitial inflammation, arteriolar hyalinosis, and arteriosclerosis), 5 pathological variables (glomerular class by the RPS classification ≥IIb, IFTA, interstitial inflammation, arteriolar hyalinosis, and arteriosclerosis), and clinical variables (age, male, eGFR, urinary protein excretion, hemoglobin A1c, hypertension, total cholesterol, use of RAAS inhibitors). The cumulative incidence for the progression of DKD was estimated using Kaplan–Meier analysis, and the statistical differences between groups by the presence or absence of polar vasculosis were examined using the log‐rank test. To determine the association of pathological variables including polar vasculosis and DKD progression among the subgroups of baseline GFR or proteinuria categories, multivariate Cox proportional hazard regression models of 13 pathological variables in the Japanese classification and 6 pathological variables were performed after adjustment for baseline clinical variables (age, male, eGFR, and urinary protein excretion). Because DKD progression and all‐cause mortality were considered competing events in this study^20^, we additionally conducted cumulative incidence function with Gray's test and the multivariate Fine‐Gray regression model (the subdistribution hazard regression model). Values of *P* <0.05 were considered statistically significant. All analyses were performed using Stata version 17.0 software (StataCorp, College Station, TX, USA) and R version 4.2.3.

## RESULTS

### Baseline characteristics

The baseline characteristics of the 811 patients included in this study are shown in Table 1. Regarding the clinical variables, the median age of the patients was 60.0 (51.0–66.0) years, and 67.8% (550/811) were male. The median serum creatinine, eGFR, and urinary protein excretion levels were 1.1 (0.8–1.6) mg/dL, 49.7 (33.4–69.7) mL/min/1.73 m^2^, and 1.9 (0.6–4.5) g/g creatinine or g/day, respectively. The proportions of G1, G2, G3a, G3b, G4, and G5 in the GFR categories were 10.7% (87/811), 24.2% (196/811), 21.7% (176/811), 24.2% (196/811), 15.2% (123/811), and 4.1% (33/811), respectively. The proportions of 0.15–0.49 g/g creatinine (g/day) and ≥0.5 g/g creatinine (g/day) in proteinuria categories were 20.5% (166/811) and 79.5% (645/811), respectively.

**Table 1:** Baseline clinical and pathological characteristics by the presence of polar vasculosis

	All	Polar vasculosis		
	Total (*n* = 811)	Absence (*n* = 134)	Presence (*n* = 677)	*P*
Clinical variables				
Age (years)	60.0 (51.0, 66.0)	60.0 (49.0, 66.0)	60.0 (52.0, 66.0)	0.460
Male, *n* (%)	550 (67.8)	95 (70.9)	455 (67.2)	0.404
Serum creatinine (mg/dL)	1.1 (0.8, 1.6)	1.2 (0.8, 1.8)	1.1 (0.8, 1.6)	0.060
eGFR (mL/min/1.73 m^2^)	49.7 (33.4, 69.7)	45.4 (29.9, 65.7)	50.3 (34.3, 70.8)	0.130
GFR categories, *n* (%)				
G1	87 (10.7)	13 (9.7)	74 (10.9)	0.478
G2	196 (24.2)	29 (21.6)	167 (24.7)	
G3a	176 (21.7)	26 (19.4)	150 (22.2)	
G3b	196 (24.2)	32 (23.9)	164 (24.2)	
G4	123 (15.2)	28 (20.9)	95 (14.0)	
G5	33 (4.1)	6 (4.5)	27 (4.0)	
Urinary protein excretion (g/g creatinine or g/day)	1.9 (0.6, 4.5)	1.9 (0.6, 4.1)	1.9 (0.6, 4.5)	0.870
Proteinuria categories, *n* (%)				
0.15–0.49 g/g creatinine (g/day)	166 (20.5)	26 (19.4)	140 (20.7)	0.738
≥0.5 g/g creatinine (g/day)	645 (79.5)	108 (80.6)	537 (79.3)	
Hemoglobin A1c (%)	7.3 (6.3, 8.8)	7.1 (6.2, 8.5)	7.4 (6.4, 8.8)	0.148
Systolic blood pressure (mmHg)	140.0 (126.0, 156.0)	143.0 (132.0, 160.0)	140.0 (126.0, 154.0)	0.077
Diastolic blood pressure (mmHg)	78.0 (70.0, 86.0)	80.0 (72.0, 90.0)	78.0 (68.0, 86.0)**	<0.001
Hypertension, *n* (%)	408 (56.3)	68 (61.8)	340 (55.3)	0.203
Total cholesterol (mg/dL)	208.0(174.5, 246.0)	209.0 (175.0, 260.0)	207.0 (173.0, 244.0)	0.400
Use of antihyperglycemic treatment, *n* (%)	366 (66.8)	34 (54.8)	332 (68.3)*	0.034
Use of RAAS inhibitors, *n* (%)	285 (51.7)	44 (63.8)	241 (50.0)*	0.032
Use of calcium channel blockers, *n* (%)	283 (51.7)	35 (52.2)	248 (51.7)	0.930
Use of lipid‐lowering agents, *n* (%)	125 (22.8)	20 (29.9)	105 (21.8)	0.143
Pathological variables				
Glomerular lesions				
Diffuse mesangial expansion, *n* (%)				
0	16 (2.0)	6 (4.5)	10 (1.5)**	<0.001
1	179 (22.1)	42 (31.3)	137 (20.3)**	
2	215 (26.5)	40 (29.9)	175 (25.9)**	
3	400 (49.4)	46 (34.3)	354 (52.4)**	
Nodular sclerosis, *n* (%)	358 (45.2)	37 (29.8)	321 (48.0)**	<0.001
Subendothelial widening of GBM, *n* (%)				
0	256 (32.3)	75 (57.7)	181 (27.3)**	<0.001
1	315 (39.7)	35 (26.9)	280 (42.2)**	
2	149 (18.8)	13 (10.0)	136 (20.5)**	
3	73 (9.2)	7 (5.4)	66 (10.0)**	
Exudative lesion, *n* (%)	420 (52.0)	50 (37.9)	370 (54.7)**	<0.001
Mesangiolysis, *n* (%)	319 (39.7)	36 (27.5)	283 (42.1)**	0.002
Percent global glomerulosclerosis	19.0 (5.6, 36.3)	20.0 (3.6, 40.0)	18.2 (5.9, 35.3)	0.406
Percent segmental glomerulosclerosis	0.0 (0.0, 0.0)	0.0 (0.0, 0.0)	0.0 (0.0, 0.0)	0.784
Glomerulomegaly, *n* (%)	304 (37.5)	33 (24.6)	271 (40.1)**	0.001
Glomerular class by the RPS classification, *n* (%)				
<IIa	12 (1.5)	5 (3.7)	7 (1.0)**	<0.001
IIa	301 (37.1)	64 (47.8)	237 (35.0)**	
IIb	86 (10.6)	15 (11.2)	71 (10.5)**	
III	294 (36.3)	24 (17.9)	270 (39.9)**	
IV	118 (14.6)	26 (19.4)	92 (13.6)**	
Tubulointerstitial lesions				
IFTA, *n* (%)				
0	51 (6.3)	19 (14.3)	32 (4.7)**	<0.001
1	298 (36.9)	31 (23.3)	267 (39.6)**	
2	224 (27.7)	35 (26.3)	189 (28.0)**	
3	235 (29.1)	48 (36.1)	187 (27.7)**	
Interstitial inflammation, *n* (%)				
0	139 (17.1)	25 (18.7)	114 (16.8)	0.527
1	391 (48.2)	59 (44.0)	332 (49.0)	
2	156 (19.2)	31 (23.1)	125 (18.5)	
3	125 (15.4)	19 (14.2)	106 (15.7)	
Vascular lesions				
Arteriolar hyalinosis, *n* (%)				
0	52 (6.4)	18 (13.5)	34 (5.0)**	<0.001
1	151 (18.6)	36 (27.1)	115 (17.0)**	
2	162 (20.0)	31 (23.3)	131 (19.4)**	
3	445 (54.9)	48 (36.1)	397 (58.6)**	
Arteriosclerosis, *n* (%)				
0	118 (15.5)	24 (19.7)	94 (14.7)	0.084
1	334 (43.9)	59 (48.4)	275 (43.0)	
2	309 (40.6)	39 (32.0)	270 (42.3)	

Data are median (interquartile range) or proportions (%). *P < 0.05 vs patients without polar vasculosis, **P < 0.01 vs patients without polar vasculosis. eGFR, estimated glomerular filtration rate; GBM, glomerular basement membrane; IFTA, interstitial fibrosis and tubular atrophy; RAAS, renin–angiotensin–aldosterone system; RPS, renal pathology society.

### Clinical and pathological characteristics by the presence of polar vasculosis

Polar vasculosis was present in 677 (83.5%) patients. Table 1 shows the baseline clinical and pathological characteristics stratified by the presence of polar vasculosis in the overall cohort. Regarding clinical variables, patients with polar vasculosis had a lower diastolic blood pressure, higher prevalence of antihyperglycemic treatment, and lower prevalence of RAAS inhibitors use. Regarding pathological variables in patients with polar vasculosis, diffuse mesangial expansion, nodular sclerosis, subendothelial widening of GBM, exudative lesion, mesangiolysis, glomerulomegaly, glomerular class by the RPS classification ≥IIb, and arteriolar hyalinosis were more advanced, whereas IFTA was less advanced.

### Pathological and clinical variables associated with the presence of polar vasculosis

To investigate the associations between polar vasculosis and other kidney lesions and baseline clinical variables, we used univariate and multivariate logistic regression analyses (Table 2). In the univariate analysis, diffuse mesangial expansion (odds ratio [OR]: 1.57; 95% CI: 1.27–1.93, *P* < 0.001), nodular sclerosis (OR: 2.17; 95% CI 1.43–3.28, *P* < 0.001), subendothelial widening of GBM (OR: 1.98; 95% CI: 1.54–2.53, *P* < 0.001), exudative lesion (OR: 1.98; 95% CI: 1.35–2.91, *P* < 0.001), mesangiolysis (OR: 1.91; 95% CI: 1.27–2.89, *P* = 0.002), glomerulomegaly (OR 2.05; 95% CI: 1.34–3.12, *P* = 0.001), glomerular class by the RPS classification ≥IIb (OR: 1.88; 95% CI: 1.30–2.74, *P* = 0.001), arteriolar hyalinosis (OR: 1.62; 95% CI: 1.35–1.94, *P* < 0.001), and arteriosclerosis (OR: 1.35; 95% CI: 1.03–1.77, *P* = 0.029) were positively associated with the presence of polar vasculosis, whereas the use of RAAS inhibitors was negatively associated with the presence of polar vasculosis (OR: 0.57; 95% CI: 0.34–0.96, *P* = 0.034). In the multivariate analysis of pathological variables, subendothelial widening of GBM (OR: 2.08; 95% CI: 1.52–2.84, *P* < 0.001), glomerulomegaly (OR: 1.81; 95% CI: 1.09–3.00, *P* = 0.022), glomerular class by the RPS classification ≥IIb (OR: 2.41; 95% CI: 1.48–3.93, *P* < 0.001), arteriolar hyalinosis (OR: 1.57; 95% CI: 1.24–1.98, *P* < 0.001 in the 12 pathological variables model, and OR: 1.65; 95% CI: 1.33–2.05, *P* < 0.001 in the 5 pathological variables model), and arteriosclerosis (OR: 1.37; 95% CI: 1.01–1.87, *P* = 0.043 in the 12 pathological variables model, and OR: 1.33; 95% CI: 1.00–1.77, *P* = 0.048 in the 5 pathological variables model) were positively associated with the presence of polar vasculosis, whereas IFTA was negatively associated with the presence of polar vasculosis (OR: 0.55; 95% CI: 0.38–0.80, *P* = 0.002 in the 12 pathological variables model, and OR: 0.62; 95% CI: 0.45–0.85, *P* = 0.003 in the 5 pathological variables model). In the multivariate analysis of clinical variables, age (OR: 1.05; 95% CI: 1.02–1.08, *P* = 0.001), eGFR (OR: 1.02; 95% CI: 1.00–1.03, *P* = 0.018), and hemoglobin A1c (OR: 1.26; 95% CI: 1.04–1.51, *P* = 0.016) were positively associated with the presence of polar vasculosis.

**Table 2:** Pathological and clinical variables associated with the presence of polar vasculosis

Variables	Univariate analysis			Multivariate analysis								
	OR	95% CI	*P*	12 Pathological variables (Japanese classification)			5 Pathological variables			Clinical variables		
				OR	95% CI	*P*	OR	95% CI	*P*	OR	95% CI	*P*
Pathological variables												
Glomerular lesions												
Diffuse mesangial expansion	1.57	1.27–1.93	**<0.001**	1.35	0.95–1.90	0.090						
Nodular sclerosis	2.17	1.43–3.28	**<0.001**	1.31	0.69–2.51	0.408						
Subendothelial widening of GBM	1.98	1.54–2.53	**<0.001**	2.08	1.52–2.84	**<0.001**						
Exudative lesion	1.98	1.35–2.91	**<0.001**	1.56	0.92–2.65	0.101						
Mesangiolysis	1.91	1.27–2.89	**0.002**	0.96	0.51–1.78	0.886						
Global glomerulosclerosis	0.99	0.98–1.00	0.121	0.99	0.98–1.01	0.302						
Segmental glomerulosclerosis	1.00	0.97–1.02	0.775	1.00	0.97–1.04	0.895						
Glomerulomegaly	2.05	1.34–3.12	**0.001**	1.81	1.09–3.00	**0.022**						
Glomerular class ≥IIb by the RPS classification	1.88	1.30–2.74	**0.001**				2.41	1.48–3.93	**<0.001**			
Tubulointerstitial lesions												
IFTA	0.94	0.77–1.15	0.531	0.55	0.38–0.80	**0.002**	0.62	0.45–0.85	**0.003**			
Interstitial inflammation	1.00	0.82–1.22	0.991	0.87	0.62–1.22	0.417	0.96	0.70–1.30	0.769			
Vascular lesions												
Arteriolar hyalinosis	1.62	1.35–1.94	**<0.001**	1.57	1.24–1.98	**<0.001**	1.65	1.33–2.05	**<0.001**			
Arteriosclerosis	1.35	1.03–1.77	**0.029**	1.37	1.01–1.87	**0.043**	1.33	1.00–1.77	**0.048**			
Clinical variables												
Age	1.01	0.99–1.02	0.196							1.05	1.02–1.08	**0.001**
Male	0.84	0.56–1.26	0.404							0.63	0.31–1.27	0.199
eGFR	1.00	1.00–1.01	0.186							1.02	1.00–1.03	**0.018**
Urinary protein excretion	1.03	0.98–1.09	0.286							1.10	0.99–1.22	0.071
Hemoglobin A1c	1.08	0.98–1.18	0.129							1.26	1.04–1.51	**0.016**
Hypertension	0.76	0.50–1.16	0.204							1.00	0.52–1.92	0.998
Total cholesterol	1.00	1.00–1.00	0.177							1.00	0.99–1.00	0.081
Use of RAAS inhibitors	0.57	0.34–0.96	**0.034**							0.77	0.41–1.43	0.405

Values in bold are statistically significant. CI, confidence interval; eGFR, estimated glomerular filtration rate; GBM, glomerular basement membrane; IFTA, interstitial fibrosis and tubular atrophy; OR, odds ratio; RAAS, renin–angiotensin–aldosterone system; RPS, renal pathology society.

### Associations between polar vasculosis and incidence of DKD progression

**Figure 1:**
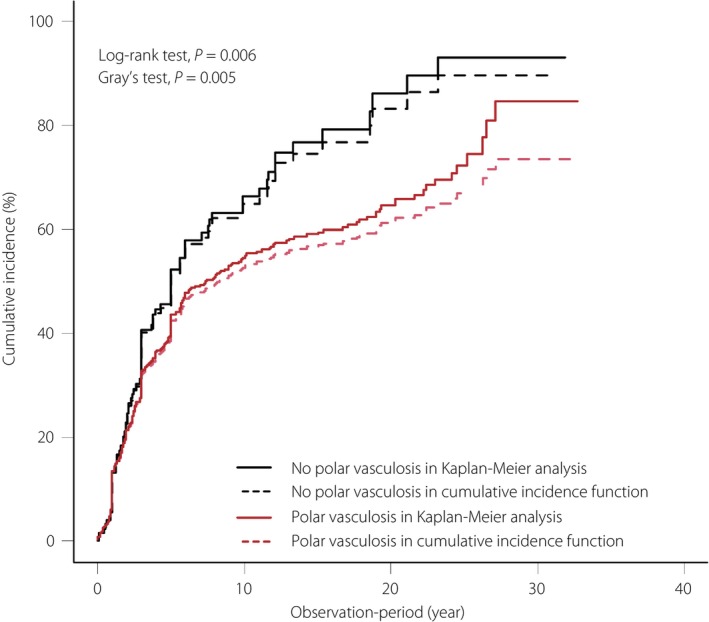
Cumulative incidence of diabetic kidney disease (DKD) progression in patients with polar vasculosis (red lines) and without polar vasculosis (black lines) in 677 and 134 patients, respectively. The solid lines are cumulative incidences calculated by Kaplan–Meier analysis. The dotted lines are cumulative incidences calculated by cumulative incidence function considering all‐cause mortality before DKD progression as a competing event.

During the median follow‐up of 5.2 years (25th–75th percentile: 2.2–10.5, maximum: 32.8), DKD progression occurred in 322 of 677 (47.6%, 7.4 events/100 person‐years) and 79 of 134 (59.0%, 11.4 events/100 person‐years) patients with and without polar vasculosis, respectively. The cumulative incidences of DKD progression stratified by polar vasculosis are shown in Figure 1. In the Kaplan–Meier analysis among the overall cohort, the probability of DKD progression was 54.5% at 10 years, 64.6% at 20 years, and 84.7% at 30 years among patients with polar vasculosis, whereas the probability of DKD progression was 66.4% at 10 years, 86.2% at 20 years, and 93.1% at 30 years among patients without polar vasculosis. The cumulative incidences of DKD progression among patients with polar vasculosis were significantly lower than those without polar vasculosis (log‐rank test, *P* = 0.006).

In the cumulative incidence functions for DKD progression by the presence of polar vasculosis in consideration of all‐cause mortality before DKD progression as a competing event, the cumulative incidence of DKD progression was lower than that of the Kaplan–Meier analysis (Figure 1). There were 59 patients who had all‐cause mortality before reaching DKD progression and 351 patients who were lost to follow‐up. The probability of DKD progression was 52.5% at 10 years, 61.1% at 20 years, and 73.7% at 30 years among patients with polar vasculosis, whereas the probability of DKD progression was 65.0% at 10 years, 83.3% at 20 years, and 89.6% at 30 years among patients without polar vasculosis. The cumulative incidences functions for DKD progression among patients with polar vasculosis were significantly lower than those without polar vasculosis (Gray's test, *P* = 0.005).

### Impact of polar vasculosis on DKD progression

To identify the association of polar vasculosis on DKD progression, we first conducted multivariate analyses (Cox and Fine‐Gray models) among the G1–G2 and G3a–G5 categories. In the Cox model including 13‐pathological variables in the Japanese classification, the presence of polar vasculosis was associated with a lower risk of DKD progression among the G1–G2 (HR: 0.38; 95% CI: 0.20–0.71, *P* = 0.003) and G3a–G5 categories (HR: 0.64; 95% CI: 0.45–0.92, *P* = 0.015; Table 3). Even in the Fine‐Gray model considering all‐cause mortality before DKD progression as a competing event, the presence of polar vasculosis was associated with a lower risk of DKD progression among G1–G2 (subdistribution HR [SHR]: 0.36; 95% CI: 0.21–0.62, *P* < 0.001) and G3a–G5 categories (SHR: 0.69; 95% CI: 0.50–0.96, *P* = 0.030; Table 3). In the Cox model including 6 pathological variables, the presence of polar vasculosis was associated with a lower risk of DKD progression among the G1–G2 (HR: 0.44; 95% CI: 0.25–0.78, *P* = 0.005) and G3a–G5 categories (HR: 0.66; 95% CI: 0.48–0.90, *P* = 0.010; Table S1). Even in the Fine‐Gray model considering all‐cause mortality before DKD progression as a competing event, the presence of polar vasculosis was associated with a lower risk of DKD progression among G1–G2 (SHR: 0.40; 95% CI: 0.24–0.66, *P* < 0.001) and G3a–G5 categories (SHR: 0.68; 95% CI: 0.50–0.91, *P* = 0.011; Table S1).

**Table 3:** Multivariate Cox and Fine‐Gray regression results for DKD progression including 13 pathological variables among subgroups stratified by GFR categories

Variables	GFR categories											
	G1–G2						G3a–G5					
	Cox model			Fine‐Gray model			Cox model			Fine‐Gray model		
	HR	95% CI	*P*	SHR	95% CI	*P*	HR	95% CI	*P*	SHR	95% CI	*P*
Pathological variables												
Glomerular lesions												
Polar vasculosis (yes/no)	0.38	0.20–0.71	**0.003**	0.36	0.21–0.62	**<0.001**	0.64	0.45–0.92	**0.015**	0.69	0.50–0.96	**0.030**
Diffuse mesangial expansion score (+1)	1.00	0.69–1.47	0.982	0.95	0.63–1.42	0.800	1.00	0.80–1.26	0.993	0.95	0.76–1.18	0.620
Nodular sclerosis (yes/no)	2.32	1.21–4.46	**0.012**	2.18	1.10–4.33	**0.026**	1.40	0.95–2.07	0.088	1.49	1.05–2.12	**0.026**
Subendothelial widening of GBM score (+1)	0.87	0.66–1.15	0.337	0.85	0.63–1.15	0.290	1.10	0.94–1.29	0.238	1.05	0.89–1.24	0.580
Exudative lesion (yes/no)	1.48	0.87–2.51	0.149	1.39	0.83–2.34	0.210	1.10	0.80–1.52	0.567	1.06	0.79–1.43	0.690
Mesangiolysis (yes/no)	1.89	0.99–3.62	0.054	1.79	0.92–3.46	0.086	1.26	0.88–1.80	0.200	1.12	0.79–1.59	0.520
Global glomerulosclerosis (+1%)	1.00	0.99–1.02	0.745	1.00	0.98–1.02	0.920	1.01	1.00–1.01	0.086	1.01	1.00–1.01	0.110
Segmental glomerulosclerosis (+1%)	1.02	0.98–1.06	0.356	1.03	0.99–1.07	0.150	1.03	1.02–1.05	**<0.001**	1.03	1.01–1.04	**0.001**
Glomerulomegaly (yes/no)	1.06	0.62–1.80	0.834	0.98	0.56–1.72	0.940	0.99	0.75–1.30	0.941	0.94	0.71–1.24	0.660
Tubulointerstitial lesions												
IFTA score (+1)	1.88	1.16–3.03	**0.010**	1.91	1.10–3.29	**0.021**	1.35	1.10–1.67	**0.005**	1.33	1.06–1.65	**0.012**
Interstitial inflammation score (+1)	0.87	0.55–1.38	0.560	0.92	0.54–1.54	0.740	0.94	0.78–1.12	0.480	0.93	0.78–1.12	0.460
Vascular lesions												
Arteriolar hyalinosis score (+1)	1.25	0.96–1.62	0.098	1.27	0.97–1.66	0.085	1.04	0.89–1.22	0.627	1.08	0.93–1.26	0.320
Arteriosclerosis score (+1)	1.10	0.80–1.52	0.558	1.07	0.80–1.44	0.640	0.95	0.77–1.16	0.619	0.94	0.77–1.15	0.560

Multivariable models were adjusted for age sex, eGFR, and urinary protein excretion. Values in bold are statistically significant.

CI, confidence interval; GBM, glomerular basement membrane; HR, hazard ratio; IFTA, interstitial fibrosis and tubular atrophy; SHR, subdistribution hazard ratio.

Next, we conducted multivariate analyses (Cox and Fine‐Gray models) among urinary protein excretion 0.15–0.49 g/g creatinine (g/day) and ≥0.5 g/g creatinine (g/day) categories. In the Cox model including the 13 pathological variables in the Japanese classification, the presence of polar vasculosis was associated with a lower risk of DKD progression among urinary protein excretion 0.15–0.49 g/g creatinine (g/day) (HR: 0.22; 95% CI: 0.07–0.74, *P* = 0.014) and urinary protein excretion ≥0.5 g/g creatinine (g/day) categories (HR: 0.67; 95% CI: 0.49–0.93, *P* = 0.015; Table 4). Even in the Fine‐Gray model considering all‐cause mortality before DKD progression as a competing event, the presence of polar vasculosis was associated with a lower risk of DKD progression among urinary protein excretion 0.15–0.49 g/g creatinine (g/day) (SHR: 0.25; 95% CI: 0.10–0.64, *P* = 0.004) and urinary protein excretion ≥0.5 g/g creatinine (g/day) categories (SHR: 0.72; 95% CI: 0.53–0.96, *P* = 0.026; Table 4). In the Cox regression model including 6 pathological variables, the presence of polar vasculosis was associated with a lower risk of DKD progression among urinary protein excretion 0.15–0.49 g/g creatinine (g/day) (HR: 0.15; 95% CI: 0.06–0.40, *P* < 0.001) and urinary protein excretion ≥0.5 g/g creatinine (g/day) categories (HR: 0.67; 95% CI: 0.51–0.89, *P* = 0.006; Table S2). Even in the Fine‐Gray model considering all‐cause mortality before DKD progression as a competing event, the presence of polar vasculosis was associated with a lower risk of DKD progression among the urinary protein excretion 0.15–0.49 g/g creatinine (g/day) (SHR: 0.13; 95% CI: 0.06–0.32, *P* < 0.001) and urinary protein excretion ≥0.5 g/g creatinine (g/day) categories (SHR: 0.67; 95% CI: 0.51–0.88, *P* = 0.004; Table S2).

**Table 4:** Multivariate Cox and Fine‐Gray regression results for DKD progression including 13 pathological variables among subgroups stratified by proteinuria categories

Variables	Proteinuria categories											
	0.15–0.49 g/g creatinine (g/day)						≥0.5 g/g creatinine (g/day)					
	Cox model			Fine‐Gray model			Cox model			Fine‐Gray model		
	HR	95% CI	*P*	SHR	95% CI	*P*	HR	95% CI	*P*	SHR	95% CI	*P*
Pathological variables												
Glomerular lesions												
Polar vasculosis (yes/no)	0.22	0.07–0.74	**0.014**	0.25	0.10–0.64	**0.004**	0.67	0.49–0.93	**0.015**	0.72	0.53–0.96	**0.026**
Diffuse mesangial expansion score (+1)	0.51	0.22–1.19	0.120	0.50	0.14–1.78	0.290	0.95	0.78–1.15	0.584	0.88	0.73–1.06	0.180
Nodular sclerosis (yes/no)	5.48	1.15–26.18	**0.033**	5.15	0.58–45.85	0.140	1.61	1.15–2.25	**0.005**	1.70	1.25–2.29	**0.001**
Subendothelial widening of GBM score (+1)	1.27	0.61–2.64	0.528	0.96	0.43–2.15	0.920	1.04	0.90–1.19	0.618	1.01	0.87–1.17	0.860
Exudative lesion (yes/no)	0.41	0.10–1.68	0.217	0.48	0.13–1.77	0.270	0.99	0.75–1.31	0.959	0.95	0.73–1.23	0.690
Mesangiolysis (yes/no)	6.04	1.28–28.54	**0.023**	5.16	0.85–31.14	0.074	1.30	0.95–1.76	0.100	1.16	0.86–1.56	0.340
Global glomerulosclerosis (+1%)	1.01	0.97–1.05	0.521	1.00	0.97–1.03	0.980	1.00	1.00–1.01	0.207	1.00	1.00–1.01	0.270
Segmental glomerulosclerosis (+1%)	1.20	1.05–1.37	**0.006**	1.21	1.02–1.44	**0.028**	1.03	1.01–1.04	**<0.001**	1.02	1.01–1.04	**0.003**
Glomerulomegaly (yes/no)	0.49	0.10–2.39	0.380	0.46	0.09–2.31	0.340	0.94	0.74–1.20	0.625	0.87	0.68–1.12	0.270
Tubulointerstitial lesions												
IFTA score (+1)	1.64	0.66–4.06	0.284	1.49	0.61–3.61	0.380	1.40	1.16–1.70	**<0.001**	1.37	1.12–1.66	**0.002**
Interstitial inflammation score (+1)	2.60	0.97–6.92	0.057	2.70	0.95–7.68	0.063	0.90	0.77–1.06	0.211	0.90	0.77–1.07	0.220
Vascular lesions												
Arteriolar hyalinosis score (+1)	1.46	0.86–2.49	0.162	1.41	0.95–2.10	0.089	1.11	0.97–1.29	0.139	1.14	0.99–1.31	0.070
Arteriosclerosis score (+1)	0.73	0.36–1.48	0.379	0.72	0.42–1.22	0.220	0.94	0.79–1.13	0.525	0.94	0.79–1.11	0.450

Multivariable models were adjusted for age sex, eGFR, and urinary protein excretion. Values in bold are statistically significant.

CI, confidence interval; GBM, glomerular basement membrane; HR, hazard ratio; IFTA, interstitial fibrosis and tubular atrophy; SHR, subdistribution hazard ratio.

In contrast, nodular sclerosis among the G1–G2 and urinary protein excretion ≥0.5 g/g creatinine (g/day) categories (Tables 3 and 4), segmental glomerulosclerosis among G3a–G5, urinary protein excretion 0.15–0.49 g/g (g/day), and urinary protein excretion ≥0.5 g/g creatinine (g/day) categories (Tables 3 and 4), glomerular class by the RPS classification ≥IIb among the G1–G2, G3a–G5, urinary protein excretion 0.15–0.49 g/g (g/day), and urinary protein excretion ≥0.5 g/g creatinine (g/day) categories (Tables S1 and S2), and IFTA among G1–G2, G3a–G5, and urinary protein excretion ≥0.5 g/g creatinine (g/day) categories (Tables 3 and 4, Tables S1 and S2) were associated with a higher risk of DKD progression in both Cox and Fine‐Gray models. Nodular sclerosis among the G3a–G5 category was associated with a higher risk of DKD progression in the Fine‐Gray model, but not in the Cox model (Table 3). Nodular sclerosis and mesangiolysis among urinary protein excretion 0.15–0.49 g/g (g/day) category were associated with a higher risk of DKD progression in the Cox model, but not in the Fine‐Gray model (Table 4).

## DISCUSSION

This multicenter study investigated a large cohort of patients with type 2 diabetes and biopsy‐proven DKD and showed two findings: (i) the presence of polar vasculosis was related to less advanced IFTA in contrast to more advanced glomerular lesions (subendothelial widening of GBM, glomerulomegaly, glomerular class by the RPS classification ≥IIb) and vascular lesions, and (ii) the presence of polar vasculosis was associated with a better kidney outcome in patients with proteinuria regardless of the subgroups stratified by GFR and proteinuria categories. We have previously shown that polar vasculosis was associated with preserved eGFR for 3 years after kidney biopsy^12^. The favorable impact of polar vasculosis on long‐term kidney outcome is consistent with our previous finding.

First, we explored the association between polar vasculosis and other kidney lesions and clinical variables. In the multivariate logistic regression analysis, IFTA was negatively associated with polar vasculosis, whereas subendothelial widening of GBM, glomerulomegaly, glomerular class by the RPS classification ≥IIb, vascular lesions, age, eGFR, and hemoglobin A1c were positively associated with polar vasculosis. Previous several studies have shown the relationships between polar vasculosis and more advanced mesangial expansion, glomerulomegaly, and arteriolar hyalinosis in human DKD, consistent with this study^5^, ^6^, ^7^, ^11^. Notably, this study also found a negative correlation between polar vasculosis and IFTA in addition to previous findings. Although the mechanism for the link between polar vasculosis and less advanced IFTA is unclear in this study, our study showed that patients with polar vasculosis did not differ in urinary protein excretion from those without polar vasculosis, despite their advanced glomerular lesions and arteriolar hyalinosis. A study of type 1 diabetes by Osterby *et al*.^5^ suggested that neovascularization at the vascular pole region may suppress the elevated urinary albumin excretion associated with prolonged diabetic glomerular damage. In the present study of type 2 diabetes, it is speculated that the decrease in intraglomerular pressure due to the formation of polar vasculosis suppressed the increase in urinary protein excretion resulting in the suppression of the development of IFTA.

Next, we showed that the presence of polar vasculosis positively correlated with eGFR and was associated with a better kidney outcome of DKD in type 2 diabetes with proteinuria. Kaplan–Meier analyses showed that polar vasculosis was associated with lower cumulative incidences of DKD progression. Cox regression analyses showed that polar vasculosis was associated with a lower risk of DKD progression in multivariate analyses, regardless of subgroups stratified by GFR and proteinuria categories. When we conducted the cumulative incidence function and the multivariate Fine‐Gray regression model in consideration of all‐cause mortality before DKD progression as a competing event, these results were unchanged. As many studies have suggested, we also identified that more advanced IFTA was associated with a poor kidney outcome in type 2 diabetes with DKD in this study^16^, ^17^, ^18^, ^21^, ^22^, ^23^, ^24^. The negative correlation between polar vasculosis and IFTA found in this study may lead to a lower risk of DKD progression, suggesting that more attention should be paid to polar vasculosis in kidney biopsy as a pathological finding associated with a better kidney outcome.

In this study, hemoglobin A1c was positively associated with the presence of polar vasculosis in multivariate logistic regression analysis. Nyumura *et al*.^9^ reported that the incidence of polar vasculosis after kidney transplantation was higher in patients with diabetes compared with those without diabetes but was not correlated with hemoglobin A1c during the observation period, suggesting some dissociations between studies derived from some factors such as the duration of diabetes. Previous studies suggest that the mechanism of angiogenesis in DKD is that hyperglycemia and hypoxia promote the production of vascular endothelial growth factor (VEGF), which stimulates the proliferation of vascular endothelial cells^8^, ^25^, ^26^, ^27^. The expression of proangiogenic and antiangiogenic factors is altered in DKD, and endothelial VEGF‐A is one of the most potent proangiogenic factors^28^, ^29^. Glomerular VEGF‐A expression has been shown to be elevated in the early stage and decreased in the advanced stage in human DKD^6^, ^30^. Experimental and clinical studies have shown that increased glomerular VEGF‐A in the early stage is probably involved in the characteristic alterations, including abnormal angiogenesis, while decreased glomerular VEGF‐A in the later stage of the disease may promote glomerular scarring^28^, ^29^. Interestingly, a recent study showed that SGLT2 inhibitor dapagliflozin promoted angiogenesis by upregulating VEGF expression, resulting in improved pathological findings and kidney function in the early‐stage diabetic nephropathy using a rat model of type 2 diabetes^31^. The mechanisms linking polar vasculosis to these biological conditions require further studies in human DKD.

Furthermore, vascular lesions and age were also positively associated with polar vasculosis by multivariate logistic regression analysis in this study. Pathologically, DKD can be complicated by nephrosclerosis as well as by traditional diabetic kidney lesions^1^, ^21^. Several studies, including our prior reports, have revealed that disproportionately advanced tubulointerstitial and vascular lesions, despite minor diabetic glomerular lesions, which denote the presence of diabetic kidney lesions as well as nephrosclerosis, were likely to be related to the development of normoalbuminuric renal insufficiency in some patients with type 2 diabetis^21^, ^32^, ^33^. The prevalence of polar vasculosis in kidney samples from age‐matched normal controls and the relationship between nephrosclerosis (with or without DKD) and polar vasculosis could be clarified in future studies.

There are several limitations in this study. First, this retrospective study included only cases that underwent kidney biopsy, which may have resulted in bias. Particularly, we included patients who underwent kidney biopsy in clinical settings, rather than using research biopsies. Therefore, there is a possibility that biases in the indications for kidney biopsy existed among participating institutions. Second, patients without proteinuria were excluded from this study because the number of observed outcomes was insufficient to perform a survival analysis. Third, we did not quantitate polar vasculosis. To understand the importance of polar vasculosis in the progression of DKD, quantitative assessment (e.g., percentage of glomeruli with polar vasculosis among all glomeruli) is also needed. Because the quantitative analysis of renal pathology is complex and time‐consuming, we were unable to investigate quantitative assessment in this study, which deals with a relatively large number of cases. Fourth, we have tried to reduce inter‐observer and inter‐institutional variability in the pathological diagnosis including polar vasculosis as a collaborative study, but the current study did not assess them in detail. Fifth, we used only baseline clinical variables in our analyses, although the changes in clinical variables and the use of RAAS inhibitors during follow‐up may affect kidney outcome. However, the study has several strengths, including biopsy‐proven DKD of a large sample collected from multiple institutions in Japan and long duration follow‐up of a maximum of approximately 30 years.

In summary, this multicenter cohort study showed that polar vasculosis of DKD was associated with less advanced IFTA and a long‐term better kidney outcome in patients with type 2 diabetes and proteinuria.

## DISCLOSURE

The authors declare no conflict of interest.

Approval of the research protocol: This study was approved by the Kanazawa University Ethics Committee [No. 2016‐460 (1204)] and all participating institutions.

Informed consent: Not required, because de‐identified data were used; patients could ‘opt out’ of participation.

Registry and the registration no. of the study/trial: N/A.

Animal studies: N/A.

## Supporting information


**Table S1** **|** Multivariate Cox and Fine‐Gray regression results for diabetic kidney disease progression including 6‐pathological variables among subgroups stratified by glomerular filtration rate categories
**Table S2 |** Multivariate Cox and Fine‐Gray regression results for diabetic kidney disease progression including 6‐pathological variables among subgroups stratified by proteinuria categories
