# Supplementary material for: Polar vasculosis is associated with better kidney outcome in type 2 diabetes with biopsy‐proven diabetic kidney disease: A multicenter cohort study
Source: J Diabetes Investig. 2023 Jul 22;14(11):1268–78. doi: 10.1111/jdi.14059 (PMC10583646; doi:10.1111/jdi.14059)
Supplement: Supplementary file 1 — Table S1 | Multivariate Cox and Fine‐Gray regression results for diabetic kidney disease progression including 6‐pathological variables among subgroups stratified by glomerular filtration rate categories Table S2 | Multivariate Cox and Fine‐Gray regression results for diabetic kidney disease progression including 6‐pathological variables among subgroups stratified by proteinuria categories [file JDI-14-1268-s001.doc]

**Supplementary Table S1.** Multivariate Cox and Fine-Gray regression results for DKD progression including 6-pathological variables among subgroups stratified by GFR categories

|  | GFR categories | | | | | | | | | | | | | | | | | | | | | | | | | | | | | | |
| --- | --- | --- | --- | --- | --- | --- | --- | --- | --- | --- | --- | --- | --- | --- | --- | --- | --- | --- | --- | --- | --- | --- | --- | --- | --- | --- | --- | --- | --- | --- | --- |
|  | G1–G2 | | | | | | | | | | | | | | |  | G3a–G5 | | | | | | | | | | | | | | |
|  | Cox model | | | | | | |  | Fine-Gray model | | | | | | |  | Cox model | | | | | | |  | Fine-Gray model | | | | | | |
| Variables | HR | (95% CI) | | | | | *P* |  | SHR | (95% CI) | | | | | *P* |  | HR | (95% CI) | | | | | *P* |  | SHR | (95% CI) | | | | | *P* |
| Pathological variables |  |  |  |  |  |  |  |  |  |  |  |  |  |  |  |  |  |  |  |  |  |  |  |  |  |  |  |  |  |  |  |
| Glomerular lesions |  |  |  |  |  |  |  |  |  |  |  |  |  |  |  |  |  |  |  |  |  |  |  |  |  |  |  |  |  |  |  |
| Polar vasculosis (yes/no) | 0.44 | ( | 0.25 | – | 0.78 | ) | **0.005** |  | 0.40 | ( | 0.24 | – | 0.66 | ) | **<0.001** |  | 0.66 | ( | 0.48 | – | 0.90 | ) | **0.010** |  | 0.68 | ( | 0.50 | – | 0.91 | ) | **0.011** |
| Glomerular class by the RPS classification ≥IIb (yes/no) | 3.53 | ( | 2.04 | – | 6.12 | ) | **<0.001** |  | 3.13 | ( | 1.85 | – | 5.30 | ) | **<0.001** |  | 2.04 | ( | 1.43 | – | 2.92 | ) | **<0.001** |  | 1.71 | ( | 1.24 | – | 2.36 | ) | **0.001** |
| Tubulointerstitial lesions |  |  |  |  |  |  |  |  |  |  |  |  |  |  |  |  |  |  |  |  |  |  |  |  |  |  |  |  |  |  |  |
| IFTA score (+1) | 1.61 | ( | 1.09 | – | 2.39 | ) | **0.018** |  | 1.62 | ( | 1.00 | – | 2.63 | ) | 0.052 |  | 1.41 | ( | 1.17 | – | 1.71 | ) | **<0.001** |  | 1.36 | ( | 1.10 | – | 1.66 | ) | **0.004** |
| Interstitial inflammation score (+1) | 0.97 | ( | 0.67 | – | 1.41 | ) | 0.891 |  | 1.02 | ( | 0.65 | – | 1.59 | ) | 0.940 |  | 0.91 | ( | 0.77 | – | 1.07 | ) | 0.250 |  | 0.91 | ( | 0.77 | – | 1.07 | ) | 0.240 |
| Vascular lesions |  |  |  |  |  |  |  |  |  |  |  |  |  |  |  |  |  |  |  |  |  |  |  |  |  |  |  |  |  |  |  |
| Arteriolar hyalinosis score (+1) | 1.20 | ( | 0.94 | – | 1.53 | ) | 0.134 |  | 1.23 | ( | 0.97 | – | 1.55 | ) | 0.090 |  | 0.97 | ( | 0.84 | – | 1.12 | ) | 0.688 |  | 1.02 | ( | 0.89 | – | 1.16 | ) | 0.830 |
| Arteriosclerosis score (+1) | 1.04 | ( | 0.77 | – | 1.40 | ) | 0.821 |  | 0.97 | ( | 0.73 | – | 1.28 | ) | 0.830 |  | 0.92 | ( | 0.76 | – | 1.12 | ) | 0.396 |  | 0.93 | ( | 0.77 | – | 1.12 | ) | 0.420 |

CI, confidence interval; HR, hazard ratio; IFTA, interstitial fibrosis and tubular atrophy; RPS, renal pathology society; SHR, subdistribution hazard ratio. Multivariable models were adjusted for age sex, eGFR, and urinary protein excretion. Values in bold are statistically significant.

**Supplementary Table S2.** Multivariate Cox and Fine-Gray regression results for DKD progression including 6-pathological variables among subgroups stratified by proteinuria categories

|  | Proteinuria categories | | | | | | | | | | | | | | | | | | | | | | | | | | | | | | |
| --- | --- | --- | --- | --- | --- | --- | --- | --- | --- | --- | --- | --- | --- | --- | --- | --- | --- | --- | --- | --- | --- | --- | --- | --- | --- | --- | --- | --- | --- | --- | --- |
|  | 0.15–0.49 g/g creatinine (g/day) | | | | | | | | | | | | | | |  | ≥0.5 g/g creatinine (g/day) | | | | | | | | | | | | | | |
|  | Cox model | | | | | | |  | Fine-Gray model | | | | | | |  | Cox model | | | | | | |  | Fine-Gray model | | | | | | |
| Variables | HR | (95% CI) | | | | | *P* |  | SHR | (95% CI) | | | | | *P* |  | HR | (95% CI) | | | | | *P* |  | SHR | (95% CI) | | | | | *P* |
| Pathological variables |  |  |  |  |  |  |  |  |  |  |  |  |  |  |  |  |  |  |  |  |  |  |  |  |  |  |  |  |  |  |  |
| Glomerular lesions |  |  |  |  |  |  |  |  |  |  |  |  |  |  |  |  |  |  |  |  |  |  |  |  |  |  |  |  |  |  |  |
| Polar vasculosis (yes/no) | 0.15 | ( | 0.06 | – | 0.40 | ) | **<0.001** |  | 0.13 | ( | 0.06 | – | 0.32 | ) | **<0.001** |  | 0.67 | ( | 0.51 | – | 0.89 | ) | **0.006** |  | 0.67 | ( | 0.51 | – | 0.88 | ) | **0.004** |
| Glomerular class by the RPS classification ≥IIb (yes/no) | 2.98 | ( | 1.13 | – | 7.84 | ) | **0.027** |  | 2.85 | ( | 1.03 | – | 7.84 | ) | **0.043** |  | 2.09 | ( | 1.53 | – | 2.86 | ) | **<0.001** |  | 1.78 | ( | 1.33 | – | 2.37 | ) | **<0.001** |
| Tubulointerstitial lesions |  |  |  |  |  |  |  |  |  |  |  |  |  |  |  |  |  |  |  |  |  |  |  |  |  |  |  |  |  |  |  |
| IFTA score (+1) | 1.83 | ( | 0.86 | – | 3.92 | ) | 0.119 |  | 1.68 | ( | 0.76 | – | 3.72 | ) | 0.200 |  | 1.40 | ( | 1.17 | – | 1.66 | ) | **<0.001** |  | 1.31 | ( | 1.09 | – | 1.58 | ) | **0.003** |
| Interstitial inflammation score (+1) | 1.07 | ( | 0.53 | – | 2.16 | ) | 0.862 |  | 1.20 | ( | 0.60 | – | 2.40 | ) | 0.610 |  | 0.87 | ( | 0.75 | – | 1.02 | ) | 0.092 |  | 0.87 | ( | 0.75 | – | 1.02 | ) | 0.086 |
| Vascular lesions |  |  |  |  |  |  |  |  |  |  |  |  |  |  |  |  |  |  |  |  |  |  |  |  |  |  |  |  |  |  |  |
| Arteriolar hyalinosis score (+1) | 1.24 | ( | 0.78 | – | 1.98 | ) | 0.360 |  | 1.27 | ( | 0.91 | – | 1.76 | ) | 0.160 |  | 1.04 | ( | 0.91 | – | 1.18 | ) | 0.578 |  | 1.07 | ( | 0.95 | – | 1.21 | ) | 0.260 |
| Arteriosclerosis score (+1) | 1.11 | ( | 0.62 | – | 1.98 | ) | 0.721 |  | 1.04 | ( | 0.60 | – | 1.80 | ) | 0.900 |  | 0.91 | ( | 0.76 | – | 1.07 | ) | 0.252 |  | 0.90 | ( | 0.77 | – | 1.07 | ) | 0.230 |

CI, confidence interval; HR, hazard ratio; IFTA, interstitial fibrosis and tubular atrophy; RPS, renal pathology society; SHR, subdistribution hazard ratio. Multivariable models were adjusted for age sex, eGFR, and urinary protein excretion. Values in bold are statistically significant.
